# The Combination of Rhythm and Pitch Can Account for the Beneficial Effect of Melodic Intonation Therapy on Connected Speech Improvements in Broca’s Aphasia

**DOI:** 10.3389/fnhum.2014.00592

**Published:** 2014-08-11

**Authors:** Anna Zumbansen, Isabelle Peretz, Sylvie Hébert

**Affiliations:** ^1^Faculty of Medicine, School of Speech Pathology and Audiology, Université de Montréal, Montreal, QC, Canada; ^2^CRBLM, Centre for Research on Brain, Language and Music, McGill University, Montreal, QC, Canada; ^3^BRAMS, International Laboratory for Research on Brain, Music, and Sound, Université de Montréal, Montreal, QC, Canada; ^4^Faculty of Arts and Science, Department of Psychology, Université de Montréal, Montreal, QC, Canada

**Keywords:** aphasia, melodic intonation therapy, treatment, speech, pitch and rhythm

## Abstract

Melodic intonation therapy (MIT) is a structured protocol for language rehabilitation in people with Broca’s aphasia. The main particularity of MIT is the use of intoned speech, a technique in which the clinician stylizes the prosody of short sentences using simple pitch and rhythm patterns. In the original MIT protocol, patients must repeat diverse sentences in order to espouse this way of speaking, with the goal of improving their natural, connected speech. MIT has long been regarded as a promising treatment but its mechanisms are still debated. Recent work showed that rhythm plays a key role in variations of MIT, leading to consider the use of pitch as relatively unnecessary in MIT. Our study primarily aimed to assess the relative contribution of rhythm and pitch in MIT’s generalization effect to non-trained stimuli and to connected speech. We compared a melodic therapy (with pitch and rhythm) to a rhythmic therapy (with rhythm only) and to a normally spoken therapy (without melodic elements). Three participants with chronic post-stroke Broca’s aphasia underwent the treatments in hourly sessions, 3 days per week for 6 weeks, in a cross-over design. The informativeness of connected speech, speech accuracy of trained and non-trained sentences, motor-speech agility, and mood was assessed before and after the treatments. The results show that the three treatments improved speech accuracy in trained sentences, but that the combination of rhythm and pitch elicited the strongest generalization effect both to non-trained stimuli and connected speech. No significant change was measured in motor-speech agility or mood measures with either treatment. The results emphasize the beneficial effect of both rhythm and pitch in the efficacy of original MIT on connected speech, an outcome of primary clinical importance in aphasia therapy.

## Introduction

Aphasia is an acquired loss or impairment of the ability to communicate by language following brain damage (usually in the left hemisphere) and is present in more than one-third of stroke survivors (Wade et al., 1986; Dickey et al., 2010). Aphasia takes multiple forms. People with Broca’s aphasia, one of the aphasic syndromes, have preserved simple verbal comprehension ability but have difficulty understanding complex syntactic sentences and, on the expressive side of language, they experience word-retrieval difficulty (i.e., anomia), grammar and syntax deficit (i.e., agrammatism), and apraxia of speech, a motor-speech disorder affecting the planning or programing of speech movements (AAN, 1994; Basso, 2003).

In its original form, Melodic Intonation Therapy (MIT, Albert et al., 1973; Sparks et al., 1974) is a formalized impairment-based approach of language rehabilitation in people with Broca’s aphasia (AAN, 1994) (see Zumbansen et al., 2014 for a synthesis of MIT variations). The particularity of MIT in comparison to other therapies for aphasia is that it trains patients to produce speech using a form of singing to facilitate their speech output. The so-called intoned-speech technique is a musical stylization of the normal speech prosody using a few pitches (usually only two, separated by a third or a fourth) and a simple rhythm (quarter and eighth notes) on a slow tempo (Sparks, 2008). The stressed syllables of words are produced with higher voice intensity on the high pitch and a quarter note, whereas the unstressed syllables are produced with lower voice intensity on the low pitch and the eighth note. Patients first learn to intone speech through a structured, intensive therapeutic protocol where they are asked to produce numerous and varied short sentences, with the help of additional facilitation techniques, such as unison production, lip-reading, hand-tapping of the rhythm, and use of formulaic phrases that are often better produced in Broca’s aphasia. Each sentence is repeated several times, first in unison with the clinician and gradually more autonomously but always with the intoned-speech technique. After a series of sessions, the last level of the program guides patients to return to a normal speech output and patients are supposed to intone speech only internally. The goal of MIT is to improve propositional speech, that is, the generative and controlled language on which people rely most to express their ideas in everyday life (Jackson, 1878; Van Lancker-Sidtis and Rallon, 2004). MIT has been rated as promising for the treatment of Broca’s aphasia (AAN, 1994). It has been studied in several efficacy studies that have reported improvements in participants’ natural connected speech (Sparks et al., 1974; Bonakdarpour et al., 2003; Schlaug et al., 2008, 2009; van der Meulen et al., 2014).

The role of the melodic elements in MIT has intrigued scientists since the very early publications of MIT and a variety of mechanisms have been proposed to explain MIT’s efficacy (reviewed in Merrett et al., 2014; Zumbansen et al., 2014). To date, however, few have been tested. The early idea in the 1970s was that musical components could engage music processing regions of the right cerebral hemisphere and that these regions could potentially take over the role of the damaged left hemisphere language regions (Berlin, 1976; Helm-Estabrooks, 1983). The right-hemisphere contribution has been the most studied aspect of MIT but has not been unanimously supported (e.g., Belin et al., 1996). In fact, language hemisphere lateralization after stroke primarily depends on individual factors, and it is still unclear if any speech and language therapy can force the lateralization of language in one hemisphere or the other during brain reorganization after stroke (Anglade et al., 2014).

The role of melodic components in MIT has otherwise been studied at the behavioral level mainly with attempts to understand how rhythm or pitch could account for the beneficial effect of MIT. In transversal studies, the rhythmic component of intoned-speech production appears to be responsible for on-line facilitation of patients’ speech accuracy (Laughlin et al., 1979; Boucher et al., 2001; Stahl et al., 2011). Longitudinal studies have used variations of MIT where only a limited set of sentences (10 to 15) is repeatedly trained (i.e., palliative variations of MIT, see Zumbansen et al., 2014) and examined if participants improved their speech accuracy in normally spoken sentences trained either with intoned speech (i.e., with rhythm and pitch), with rhythmic speech (i.e., without musical pitch) or non-trained (Wilson et al., 2006; Stahl et al., 2013). Significant improvement was obtained for trained sentences compared to non-trained items and pitch did not add any beneficial effect over rhythm on speech accuracy immediately post-treatment. Therefore, the utility of pitch in MIT is currently questioned. In both studies, no transfer of improvements to the non-trained phrases was observed. One possible explanation is that these versions of MIT did not include a basic characteristic of the original MIT, namely the numerous sentences that have to be presented to avoid the use of rote memory (Sparks, 2008), a strategy that was pointed as a generalization factor by several authors (Thompson, 1989; Nadeau et al., 2008). Changes in natural connected speech, the ultimate goal of MIT, were not assessed in these studies.

For a long time, many studies have measured treatment efficacy on trained material only (e.g., number of correct syllables produced in sentences repeatedly trained). Others have used verbal tasks with non-trained items such as sentence repetition and picture naming to capture improvement in specific speech and language abilities (Brady et al., 2012). However, these tasks may not reveal how patients use language in natural speech. In reviewing efficacy studies in the aphasia literature, Beeson and Robey (2006) have distinguished direct effects on trained stimuli, generalization to non-trained stimuli, and generalization to connected speech. Here too we will refer to these effects as direct effect, indirect effect, and generalization, respectively. A common way to measure connected speech improvements in functional communication is to count the presence of Correct Information Units (CIU) in a speech sample. Nicholas and Brookshire (1993) define CIUs as words that are intelligible in context and accurately convey information relevant to the eliciting stimulus. Informativeness, the efficiency in conveying and transmitting correct information to the listener, can be calculated by dividing the number of CIUs in a speech sample by the number of words in the sample. This measure has been validated to assess language in the connected speech of people with aphasia and healthy individuals (Nicholas and Brookshire, 1993).

Little is known about the mechanisms that promote generalization to connected speech in aphasia therapy. A number of treatment components are thought to play a role in this effect (see Frey, 2013 for a recent literature and expert panel review), but to our knowledge, none has been explicitly tested as a generalization mechanism to natural discourse in impairment-based aphasia treatments. Studies on therapeutic protocols such as MIT that were designed to elicit improvements in connected speech can give insights in treatment factors promoting this type of generalization. Interestingly, the melodic characteristics of MIT, which set this treatment apart from other speech and language therapies, seem to play a role in MIT’s generalization effect. In a study with two participants with Broca’s aphasia, Schlaug et al. (2008) compared the original MIT with a control treatment differing from MIT only by the absence of the pitch and rhythmic components. MIT led to greater improvement than the non-musical treatment on measures including informativeness of connected speech. The melodic components were deemed key efficacy factors for MIT. A firmer conclusion is anticipated with the results of an ongoing randomized control trial comparing the two treatments on language outcome in connected speech (Schlaug and Norton, 2011).

Our study aims to assess the relative contribution of the rhythmic and pitch features of MIT’s generalization effect to connected speech. Thus, we designed a variation of MIT (hereafter referred to as melodic therapy) that includes basic characteristics thought to promote generalization (large number of various sentences and intensive treatment delivery). We compared this melodic therapy (MT) with two control treatments: rhythmic therapy (RT), that is, MT without musical pitch, and spoken therapy (ST), without pitch or rhythmic aspects. Furthermore, in order to capture the degrees of direct and indirect effects elicited by the melodic components, we measured speech accuracy in a subset of 10 sentences that were repeatedly trained at each treatment session and in 10 non-trained sentences.

Other proposed mechanisms related to the melodic aspect of MIT have never been assessed. One of them is that singing could keep patients motivated to continue with an intensive therapy regimen because it is a pleasurable activity (Racette et al., 2006). Data demonstrating that music and singing can positively influence mood in healthy individuals and in various clinical populations has also led to the suggestion that the singing aspect of MIT could benefit patients’ mood (Merrett et al., 2014). Finally, we have suggested that MIT could mostly benefit apraxia of speech, the motor-speech symptom of Broca’s aphasia’s syndrome (Zumbansen et al., 2014). Indeed, the best responders to MIT have this symptom in common. In a first attempt to evaluate these suggested mechanisms, we tested the mood and the motor-speech agility of the participants as additional, secondary outcomes.

## Materials and Methods

### Participants

Three native French-speaking, right-handed men with aphasia (FL, FS, and JPL) participated in the study. They were recruited through an association of persons with aphasia located in the greater Montreal area. Each had experienced a single ischemic unilateral left hemisphere cerebrovascular accident more than 1 year prior to their involvement in the study and had been through the standard public rehabilitation services, which commonly discharge aphasic patients when their language improvements reach a plateau. They had not received any speech-language therapy since. None of the participants had experienced neurological or psychiatric problems before the stroke. An examination by a certified audiologist attested that they had no hearing deficit. Table 1 summarizes patients’ characteristics, and Table 2 presents the scores of francophone language tests and non-verbal cognitive tests. Each subject had a clinical profile consistent with Broca’s aphasia, that is, naming deficits, agramatism, apraxia of speech, and relatively preserved simple verbal comprehension compared to expressive difficulties. FL and JPL had a moderate degree of aphasia whereas FS had a more severe clinical profile, especially because he experienced more severe apraxia of speech than the other participants in connected speech. FL and FS had a right upper-limb hemiplegia while JPL had almost completely recovered from it. The three participants had been treated for focal epilepsy and JPL has also been treated for depression since his stroke. All three participants were good candidates for MIT according to the American Academy of Neurology (1994): they had Broca’s aphasia and were willing to undergo intensive individual speech and language treatment. They gave their informed consent and the study was approved by the Ethical Committee of the Montreal University Geriatric Institute.

**Table 1:** **Participants’ characteristics**.

Participant	Sex	Age	Education (in years)	Years of formal musical training	Months post- stroke (at recruitment)	Aphasia diagnosis
FL	M	57	17	0	20	Moderate Broca’s aphasia
FS	M	50	13	0	24	Severe Broca’s aphasia
JPL	M	48	16	0	21	Moderate Broca’s aphasia

**Table 2:** **Participants’s language and non-verbal cognitive diagnostic assessments**.

	FL	FS	JPL
MT-86 aphasia battery (Nespoulous et al., 1992)			
Expression			
Naming/31	**17** [28]	**8** [28]	**17** [23]
Narrative discourse/18	**9** [9]	**2** [9]	**7** [8]
Global reduction of fluency	**Moderate**	**Severe**	**Moderate**
Agrammatism	**Severe**	**Severe**	**Severe**
Syntactic deviations	**Moderate**	**Severe**	**Moderate**
Anomia	**Moderate**	**Severe**	**Moderate**
Phonetic deviations	**Moderate**	**Severe**	**Moderate**
Phonemic deviations (and/or jargon)	**Moderate**	**Severe**	**Moderate**
Semantic deviations	**Moderate**	**Mild**	**Mild**
Repetition/30	**16** [24]	**9** [24]	**12** [27]
Comprehension/47	**24** [40]	**33** [40]	**34** [39]
Words/9	9	8	9
Sentences/38	**15**	**25**	**25**
Verbal fluency test (Cardebat et al., 1990)			
Phonemic fluency	**2** (−2.6)	**8** (−2.2)	**8** (−2.2)
Semantic fluency	**6** (−3.9)	**7** (−3.7)	**23** (−2.3)
Abbreviated MBEMA (Peretz et al., 2013)			
Pitch/20	15	**9** (−4.1)	14
Rhythm/20	17	16	17
Memory/20	15	**13** (−3.6)	17
PEGV (Agniel et al., 1992)			
Visual agnosia/66	62	62	66
WAIS (Wechsler, 1997a)			
Matrix reasoning/26	22	11	23
WMS (Wechsler, 1997b)			
Spatial span/32	10	15	17
Tower of London – Drexel University (Culbertson and Zillmer, 2001)			
Total move score	7	32	20

*When available, the maximum score is indicated next to the test name. Measures considered below the relevant norms for patient’s demographics are printed in bold and number of standard deviations (SD) to the mean is indicated next to the scores in parentheses. Cut-off scores for patients’ age and education are in square brackets. MBEMA, Montreal Battery of Evaluation of Musical Abilities; PEGV, Protocole d’évaluation des gnosies visuelles (Visual agnosia diagnostic battery); WAIS-III, Wechsler Adult Intelligence Scale – third edition; WMS, Wechsler Memory Scale – third edition*.

### Verbal material

A total of 240 2- to 8-syllable-long phrases were created by two graduate students in speech and language pathology and the first author (an experienced speech and language therapist). Phrases were selected so as to fit participants’ daily living, as would do a typical clinician in aphasia therapy. They were split into 180 New-phrases (2- to 8-syllable long) and 60 Test-phrases (4- to 5-syllable long). The 180 New-phrases were used for the purpose of the interventions. The same 180 sentences were used in the same order for the three consecutive treatments, so that one phrase was presented once for each treatment, leaving a minimal interval of 6 weeks between two presentations. Test-phrases served to assess the direct and indirect effects of the treatments. These items were four- to five-syllable long, that is, of medium difficulty compared to the New-phrases.

**Figure 1:**
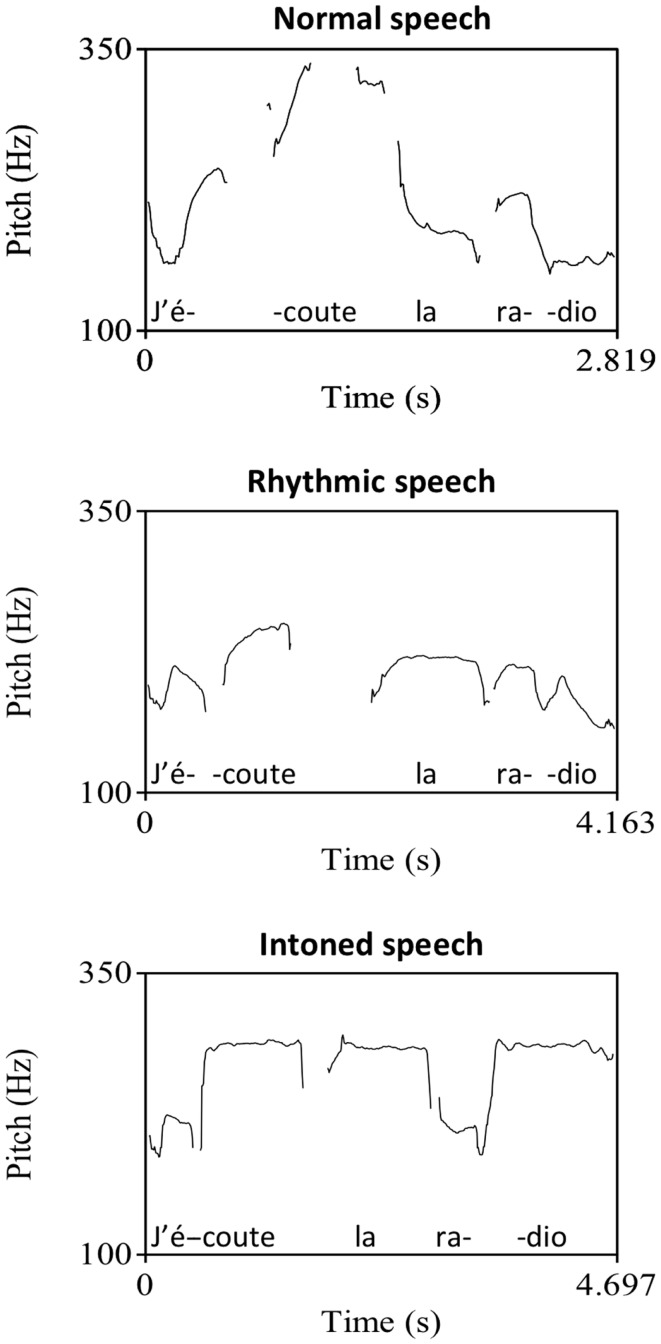
**Sentence example (“I listen to the radio”) in normal, rhythmic, and intoned speech**. Graphs represent the fundamental frequency of the voice in recordings as extracted by the software for speech analysis, Praat (Boersma and Weenick, 2013).

All sentences were recorded in three modes: intoned, rhythmically spoken, and normally spoken, for a total of 720 recordings (see an example in Figure 1). The stimuli were produced by a natural voice in the way a speech and language therapist would do in a real clinical setting following the instructions of the different production modes and with the help of pitch and tempo cues given prior to the recordings. In the intoned mode, the stimuli had pitch variation on two notes separated by a fourth interval. We chose G# and C# according to an estimate of participants’ vocal speech range to allow them to reproduce the pitches without vocal strain. Stimuli were presented by a female voice and were reproduced one octave lower by participants. Each syllable had to be produced on a single pitch. The high pitch was associated with syllables that are stressed in natural prosody of French (e.g., the last syllable of a clause) and with the syllables of function words (e.g., prepositions, pronouns, and articles), according to a French adaptation of MIT (Therapie Mélodique et Rythmée, Van Eeckhout and Bhatt, 1984), because they are often omitted in Broca’s aphasic speech. In addition to musical pitch variation, the intoned sentences were produced with rhythm: syllables had to be temporally organized on a regular beat of 100 bpm with high pitch twice as long as low pitches. In the rhythmically spoken mode, phrases had to be produced only with the rhythmic element following the same tempo cue as in the intoned mode and otherwise with continuous voice frequency variation typical of speech. In the normally spoken mode, both above mentioned pitch and rhythm elements were absent. The sentences were produced with clear and slow articulation and with prosody consistent with the French morpho-syntactic rules, as would do clinicians in standard aphasia therapy. Mean syllable duration was computed by dividing each stimulus duration in milliseconds by its number of syllables. Significant differences were found across stimuli modes. In average, compared to melodic syllables (M = 1130, SD = 146), rhythmic syllables (M = 1040, SD = 146) were 90 ms shorter while spoken syllables (M = 556, SD = 94) were twice shorter.

### Treatments

Each treatment (MT, RT, and ST) was administered by a trained graduate student in speech and language therapy, at a frequency of 3 one-hour sessions/week for 6 weeks (i.e., 18 sessions/treatment). They differed only with regard to the presence or absence of hand-tapping and musical elements in the stimuli. In the MT, patients had to repeat intoned sentences and were guided to tap the rhythm along with their left hand (hereafter simply referred to as hand-tapping). The RT consisted of rhythmically spoken stimuli and hand-tapping. In the ST, patients were presented with normally spoken stimuli and no hand-tapping was elicited.

During sessions, participant and therapist sat facing each other at a table in a quiet room. Participants had to listen and produce 20 phrases (see examples in Table 3), each following a progressive procedure in four steps: two times in unison, two times in unison with therapist fading-out at half-way, one time in repetition alone, and finally alone in response to a question. Half of the sentences were New-phrases ranging from two to eight syllables (one phrase of two, three, seven, and eight syllables and two of four, five, and six syllables), beginning with the shortest and progressing on to the longest sentences. The other half were Test-phrases repeatedly trained at each session to ultimately assess the direct effect of the treatment. The stimuli were first heard from an iPod connected to speakers and immediately reproduced by the therapist to allow lip-reading. Up to four attempts were allowed in the steps where unison was used. If the participant still failed to produce the phrase successfully, the item was discontinued and the next phrase was presented. When errors occurred at the two last steps, the preceding step was reintroduced before trying again and if this second attempt failed, the item was discontinued.

**Table 3:** **Characteristics and examples of sentences worked out during a treatment session**.

Presentation order	Item status	Number of syllables	Sentences
1	New	2	Parfait (*All right*)
2	New	3	Il fait froid (*It’s cold*)
3	New	4	Je te regarde (*I’m watching you*)
4	New	4	Prends le courrier (*Take the mail!*)
5	New	5	La porte est ouverte (*The door is open*)
6	New	5	Voici mon adresse (*Here is my address*)
7	New	6	Donne-moi de tes nouvelles (*Give me some news of you*)
8	New	6	J’écoute de la musique (*I listen to music*)
9	New	7	Dis bonjour à ta famille (*Say hi to your family*)
10	New	8	Je n’ai pas fini de manger (*I have not finished eating*)
11	Trained at each session	4	À la prochaine (*See you later*)
12	Trained at each session	4	Bon appétit (*Enjoy your meal*)
13	Trained at each session	4	Ça me fait mal (*It hurts*)
14	Trained at each session	4	Combien ça coûte (*How much is it*)
15	Trained at each session	4	Prends soin de toi (*Take care*)
16	Trained at each session	5	J’ai de la visite (*I have visitors*)
17	Trained at each session	5	J’ai un rendez-vous (*I have an appointment*)
18	Trained at each session	5	Je ne comprends pas (*I don’t understand*)
19	Trained at each session	5	Je ne viendrai pas (*I won’t come*)
20	Trained at each session	5	Pouvez-vous m’aider (*Could you help me*)

### General procedure

The study took place at the aphasic association where participants were recruited. We followed a Latin cross-over design and used the random number generation function of Microsoft Excel to allocate participants to treatment sequences: FL underwent the treatment sequence MT–RT–ST, FS underwent RT–ST–MT and JPL followed the order ST–MT–RT. Evaluations were conducted before and after each treatment phase, for a total of four evaluation periods, hereafter referred to as T1, T2, T3, and T4. Moreover, performance was measured three times within each evaluation period (T1a, T1b, T1c; T2a, T2b, T2c; T3a, T3b, T3c; T4a, T4b, T4c), with a minimum of 2-day intervals between assessments, to ensure that results would not be biased by day-to-day variations in participants’ general state. One list of 20 Test-phrases was used for each intervention phase. They were split into 10 stimuli to be repeatedly trained at each treatment session and 10 non-treated stimuli and were counterbalanced between participants.

### Assessment of treatment outcomes

Language outcomes were assessed through the repetition of trained and non-trained stimuli (direct and indirect treatment effects) and in connected speech (generalization effect) elicited in a picture description task. Motor-speech ability and mood were assessed with adapted standardized test (see below). All the assessments were videotaped and verbal performance was transcribed in order to be analyzed by a different person than the therapist.

The primary outcome was the change from pre- to post-treatment in discourse informativeness (in percent CIU in connected speech). Speech samples were elicited in a description task of 15 complex line drawing pictures of several characters acting in daily situations. This number is well above the recommended minimum number of stimuli (5) (Brookshire and Nicholas, 1994) to ensure adequate test–retest stability of informativeness in people with aphasia. Moreover, in order to control for day-to-day variations within each evaluation period, the pictures were split into three groups of five to collect speech samples on three different days. Informativeness was scored with the help of the software, Cordial Analyseur (Synapse-Développement, 2010), for words’ counts.

Secondary outcomes were the changes from pre- to post-treatment in number of correct syllables in the trained and non-trained sentences. The productions were obtained in a repetition task of the audio-recorded Test-phrases in the normally spoken mode. No lip-reading was possible. Correct syllables were rated with 1 point and syllables with an error on a single phoneme were given 0.5 point, following the procedure of Racette et al. (2006).

In a first attempt to monitor changes in apraxia of speech with MIT and in absence of a validated test in French, we chose the Diadochokinetic rate subtest of the Apraxia battery for adults (ABA2, Dabul, 2000), the best validated diagnostic battery currently available. The task consists of rapid repetitions of syllables to assess motor-speech agility. We used the total score of this ABA2 subtest.

Finally, we assessed participants’ mood with the visual analog mood scales (VAMS, Stern, 1997). On each scale, drawings of two faces are connected with a vertical 10-cm line. One face has a neutral expression while the other represents a mood state (afraid, confused, sad, angry, energetic, tired, happy, or tense). Participants have to mark on the line how they feel. This test is particularly well adapted to patients with aphasia since it requires minimal verbal abilities. T-scores on the eight mood subscales of the VAMS served for this secondary outcome.

## Results

### Primary outcomes – generalization effects to language in connected speech

**Figure 2:**
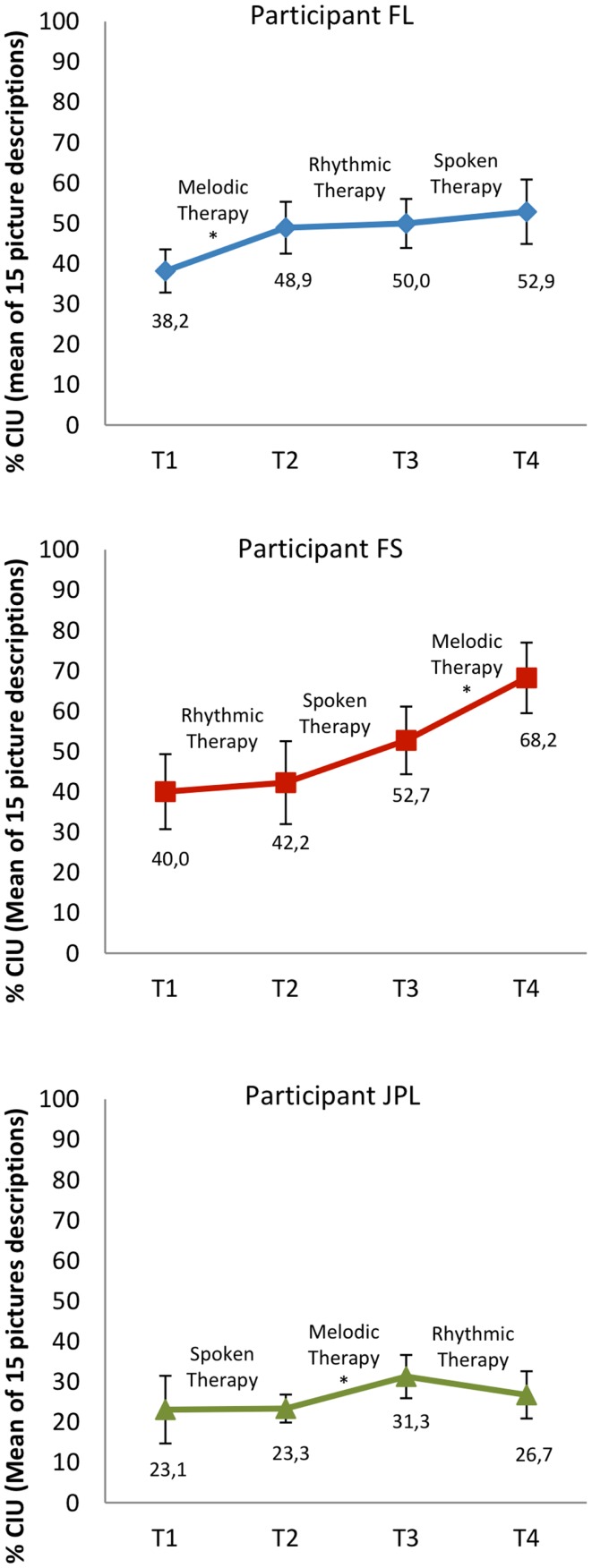
**Informativeness in connected speech at each assessment time (T1–T4), before and after treatments (i.e., generalization effects)**. Error bars represent 95% confidence intervals. The star indicate pre–post-treatment differences in non-parametric statistical tests when *p* < 0.05. CIU: Correct information units.

Participants were considered as single cases (Figure 2). We compared participants’ mean informativeness score computed from the 15 picture descriptions before and after each treatment. In FL, the Wilcoxon signed-rank tests revealed a significant progression only from T1 to T2, that is, with MT (*Z* = −2.101, *p* = 0.036). In FS, there was a significant improvement only from T3 to T4, with MT (*Z* = −2.017, *p* = 0.044). In JPL, significant change was only found from T2 to T3, with MT again (*Z* = −2.329, *p* = 0.024). In sum, in all three participants, MT had a significant generalization effect in terms of informativeness in connected speech while RT and ST had not.

### Secondary outcomes

#### Direct and indirect treatment effects

**Figure 3:**
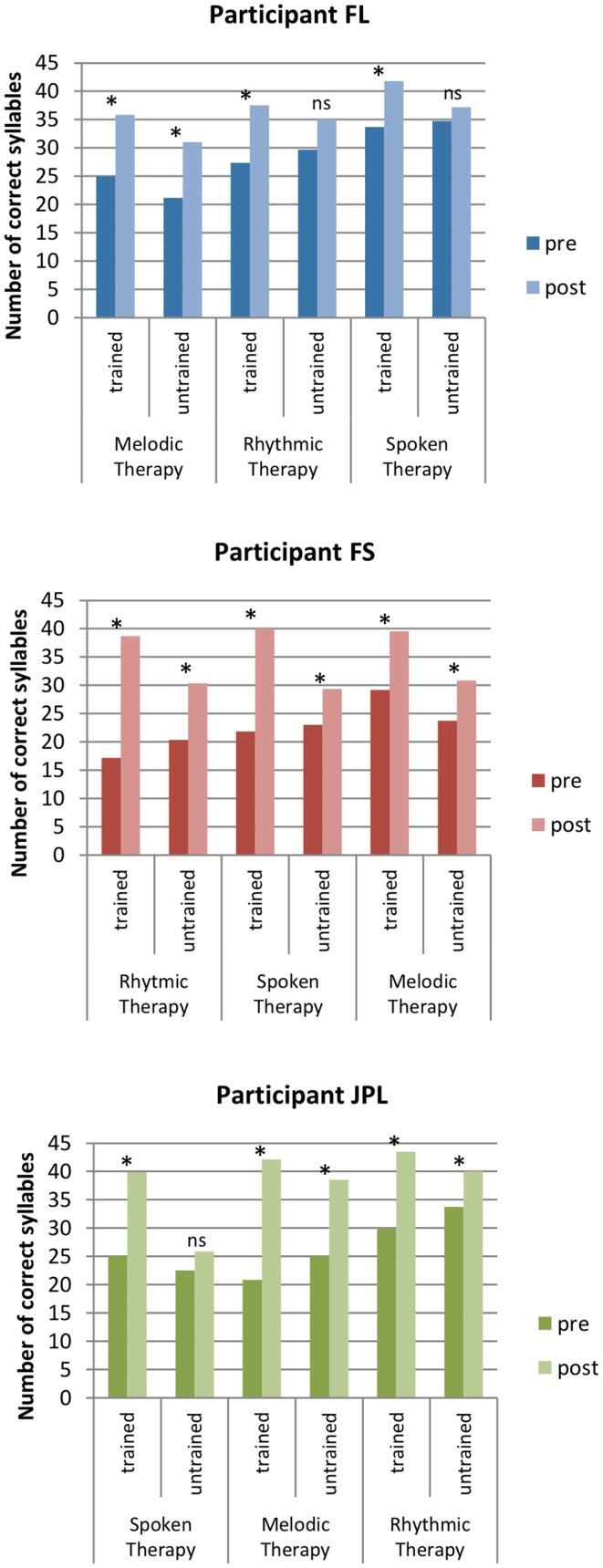
**Speech accuracy in trained and non-trained Test-phrases before and after each treatment (i.e., direct and indirect effects)**. Columns represent the total number of correct syllables in 10 phrases produced in a normally spoken repetition task. The star indicate pre–post-treatment differences in non-parametric statistical tests when*p* < 0.05.

Test-phrases were repeatedly assessed at three different days (a, b, and c) within each evaluation period before and after treatments (Table 4 and Figure 3). A preliminary analysis with Friedman tests revealed no significant difference between the repeated assessments of each list of Test-phrases within the evaluation periods of each participant. Thus, the measures appeared to be stable before or after treatments and we compared pre- to post-treatment data with Wilcoxon tests based on the mean scores of the three repeated assessments of each treated (tr) and non-treated (ntr) Test-phrase.

**Table 4:** **Mean number of correct syllables per test-phrases (*n* = 10) before and after each treatment**.

Treatment	Participant	Trained				Non-trained				Comparison of increases
		Pre-	Post-	Pre-post comparison	Increase	Pre-	Post-	Pre-post comparison	Increase	
Melodic therapy	FL	2.50 (1.00)	3.58 (0.91)	*	1.09 (1.44)	2.12 (1.08)	3.10 (0.76)	*	0.98 (0.84)	NS
	FS	2.92 (0.88)	3.95 (0.75)	*	1.03 (0.68)	2.38 (0.81)	3.08 (0.84)	*	0.71 (0.78)	NS
	JPL	2.08 (0.85)	4.22 (0.69)	*	2.13 (1.05)	2.50 (0.73)	3.85 (0.64)	*	1.35 (0.54)	NS
Rhythmic therapy	FL	2.73 (0.79)	3.75 (0.55)	*	1.02 (0.97)	2.97 (1.01)	3.50 (0.81)	NS	0.53 (1.35)	Trained > non-trained
	FS	1.72 (0.75)	3.87 (0.85)	*	2.15 (0.72)	2.03 (0.61)	3.03 (0.68)	*	1.00 (0.66)	Trained > non-trained
	JPL	3.00 (0.92)	4.35 (0.69)	*	1.35 (0.66)	3.38 (0.61)	4.00 (0.36)	*	0.63 (0.70)	Trained > non-trained
Spoken therapy	FL	3.37 (0.54)	4.18 (0.56)	*	0.82 (0.82)	3.48 (1.02)	3.72 (0.71)	NS	0.24 (1.02)	Trained > non-trained
	FS	2.18 (1.03)	4.00 (0.67)	*	1.82 (0.96)	2.30 (0.81)	2.93 (0.77)	*	0.63 (0.78)	Trained > non-trained
	JPL	2.51 (0.54)	3.98 (1.07)	*	1.47 (1.05)	2.25 (0.88)	2.58 (1.02)	NS	0.33 (1.29)	Trained > non-trained

Standard deviations (SD) are indicated in parentheses. *Significant differences in non-parametric statistical tests when *p* < 0.05.

In FL, the number of correct syllables in trained Test-phrases improved significantly with all treatments (MT[T1–T2]tr: *Z* = −2.040, *p* = 0.041; RT[T2−T3]tr: *Z* = −2.431, *p* = 0.015; ST[T3−T4]tr: *Z* = −2.134, *p* = 0.033). The production of non-trained Test-phrases also improved significantly with MT (MT[T1−T2]ntr: *Z* = −2.383, *p* = 0.017) but not with RT or ST (RT[T2−T3]ntr: *Z* = −1.023, *p* = 0.306; ST[T3−T4]: *Z* = *−*0.178, *p* = 0.859). Because there were improvements both in trained and non-trained phrases with MT, we seek to determine if speech accuracy better improved on trained versus non-trained items with this therapy. We computed for each phrase the gain in number of syllables from pre to post MT, and we compared the mean syllable gain on trained stimuli with the mean syllable gain on non-trained stimuli. We found no significant difference between the two progressions (MT[T1–T2]tr–ntr: *Z* = −0.153, *p* = 0.878).

In FS, the number of correct syllables improved significantly with all treatments in trained and non-trained Test-phrases (MT[T3–T4]tr: *Z* = −2.666, *p* = 0.008; RT[T1–T2]tr: *Z* = −2.810, *p* = 0.005; ST[T2–T3]tr: *Z* = −2.668, *p* = 0.008; MT[T3–T4]ntr: *Z* = −2.245, *p* = 0.025; RT[T1–T2]ntr: *Z* = −2.809, *p* = 0.005; ST[T2–T3]: *Z* = −2.040, *p* = 0.041). The progression was significantly greater on trained phrases than non-trained phrases following RT or ST (RT[T1–T2]tr–ntr: *Z* = −2.398, *p* = 0.016; ST[T2–T3]tr–ntr: *Z* = −2.191, *p* = 0.028) but not with MT (MT[T3–T4]tr–ntr: *Z* = −0.833, *p* = 0.405).

In JPL, there was also a significant improvement on trained Test-phrases with all treatments (MT[T2–T3]tr: *Z* = −2.703, *p* = 0.007; RT[T3–T4]tr: *Z* = −2.807, *p* = 0.005; ST[T1–T2]tr: *Z* = −2.553, *p* = 0.011). Furthermore, the production of non-trained Test-phrases also improved significantly with MT and RT (MT[T2–T3]ntr: *Z* = −2.807, *p* = 0.005; RT[T3–T4]ntr: *Z* = −2.383, *p* = 0.017) but not with ST (ST[T1–T2]ntr: *Z* = −0.866, *p* = 0.386). The progression was significantly greater on trained phrases than non-trained phrases following RT (RT[T3–T4]tr–ntr: *Z* = −2.091, *p* = 0.037) but not with MT (MT[T2–T3]tr–ntr: *Z* = −1.614, *p* = 0.107).

In sum, all treatments had a significant direct effect in each participant. The indirect effect of MT was also significant and no weaker than its direct effect, while RT had a significant indirect effect in two of three participants and was weaker than its direct effect. In only one participant, ST had a significant indirect effect and it was weaker than the direct effect.

#### Measure of motor-speech agility

We used the published norms to determine if changes on the Diadochokinetic score were significant within and between evaluation periods (Dabul, 2000). No significant variation appeared in any participant, for any treatment according to the norms.

#### Mood

The participants scored within the norms at the eight-mood subscales of the VAMS (Stern, 1997), and there was no significant variation (i.e., more than 20 *T*-score points) during the study.

## Discussion

Our primary goal was to assess the relative contribution of rhythm and pitch in MIT’s generalization effect by comparing three treatments (MT, RT, and ST) differing only by the presence or absence of these two melodic features. Only the MT, which had both pitch and rhythm, had a significant effect on the informativeness of connected speech in the participants regardless of the treatment order. Furthermore, all three forms of therapies led to improvements on trained sentences (direct effect) but their capacity to generalize these gains to non-trained sentences (indirect effect) varied. The MT showed an effect on non-trained material that was as large as the direct effect. In the other treatments, the indirect effect, when significant, was weaker than the direct effect. Finally, the presence of rhythm (in RT) had an indirect effect in two of the three participants, whereas the treatment with no melodic elements (ST) was associated with indirect effect in only one participant.

The findings show that MT was the most effective in terms of generalization effects. It replicates the results of Schlaug et al. (2008, 2009) who found better language improvements in the connected speech of one participant with MIT compared to a control therapy that did not use the musical components. With three additional participants with Broca’s aphasia, our study further supports that the combination of rhythm and pitch is valuable to language recovery in MIT. Furthermore, we found that the addition of musical pitch to the rhythmic element was associated with generalization effect to connected speech, whereas the use of rhythm only did not.

The finding of indirect effects in all participants with MT and in two participants with RT is in apparent contradiction with the results of the two previous longitudinal studies investigating the differential role of rhythm and pitch in MIT. Stahl et al. (2013) showed improvements in trained phrases but not on non-trained stimuli in two groups of subjects who underwent a melodic or a rhythmic treatment. In a controlled single case study, Wilson et al. (2006) also found significant changes in phrases trained with intoned speech or with rhythmic speech, but not in non-trained verbal material. However, none of these participants was presented with diverse New-phrases during treatment sessions that are supposed to promote generalization in original MIT (Sparks, 2008; Zumbansen et al., 2014). In fact, in the study of Stahl et al., the control group of participants who were allocated to standard therapy improved on non-trained phrases. The standard therapy consisted of a wide range of language tasks and verbal stimuli. As stressed by several authors and expert panels, the variety of verbal tasks, stimuli, and contexts may well be a key factor in the generalization effect of a speech and language therapy approach (Thompson, 1989; Nadeau et al., 2008; Frey, 2013).

One important question is to understand how pitch and rhythm, when combined, lead to some generalized language improvements. Pitch processing engages right-lateralized cerebral activity (Peretz and Zatorre, 2005), while rhythm and temporality in simple singing has been associated with left hemispheric areas that are close to language centers (Jungblut et al., 2012). So far, better language recovery has been reported with the recruitment of left perilesional cortex rather than interhemispheric compensation in post-stroke aphasia (Heiss et al., 1999; Rosen et al., 2000; Heiss and Thiel, 2006; Anglade et al., 2014). Because intoned speech engages left perilesional areas to a greater extent than normal speech in participants with aphasia after stroke (Laine et al., 1994; Belin et al., 1996), one could hypothesize that rhythm in intoned speech could be responsible for this left-hemispheric activation, leaving the pitch component as relatively unnecessary. However, in light of our behavioral results, we suggest that pitch could act as a facilitator to effectively get access to reactivation of perilesional areas for language production. Pitch information adds a redundant cue to rhythmicity in the intoned-speech technique; the high pitch is produced on the stressed syllables, which are also pronounced on the longer note, while the low pitch is on the unstressed syllables and shorter notes. We propose that pitch changes could help processing the rhythmic patterns and bootstrapping the reactivation of rhythm- and language-related left-hemispheric areas, possibly through transcallosal pathways following the classical Hebbian axiom “neurons that fire together wire together”. More brain imaging studies are clearly needed to better understand the brain correlates associated with the beneficial effect of pitch and rhythm combination on generalized language recovery after stroke. It is most plausible that the brain mechanisms of MIT vary depending on individual factors, such as the lesion size and location. In this regard, longitudinal brain imaging data from two studies with original MIT have shown increased right-hemisphere activation and white matter plasticity in nine patients with large left hemisphere lesions (Schlaug et al., 2008, 2009) and Schlaug et al. (2009) have argued that using the right hemisphere for language processing might be the only option for language improvements in such patients. When reactivation of left language areas is not possible, the pitch element of MIT could be even more crucial.

Although the three participants of our study had quite similar clinical and demographic profiles, individual differences can not easily be ruled out in clinical studies. Among the participants, FS had the lowest level of education, the most severe aphasia, and he scored lower in reasoning, planning, and musical abilities, particularly pitch processing (see the subtest of the WAIS, the Tower of London, and the Abbreviated MBEMA in Table 2). FS had theoretically more room for improvement, whereas FL and JPL were probably closer to a plateau. This could explain why FS showed indirect effects with all treatments and benefited most from the MT. Interestingly, FS did improve with MT despite his low musical abilities, suggesting that severely affected patients without good musical abilities can still benefit from pitch and rhythm combination in MIT. Melodic aspects probably affect such patients differently than patients with preserved musical skills. Turning our attention on therapists, we speculate that the use of both melodic components in MIT (compared to rhythm only) could also better entrain the clinician in a favorable attitude toward the patient to facilitate speech production during sessions, by synchronizing all facilitation techniques (unison production, lip-reading, and hand-tapping) and by enhancing the common focus of both patient and therapist. Future studies could explore the impact of melody on the therapist engagement during therapy sessions, a point of view rarely addressed in speech and language therapy.

We did not find support here for the suggestion that the musical elements of MIT would improve patient’s mood and motivation. We did not capture any mood changes that were significant according to the norms of the VAMS (Stern, 1997), whether participants were pharmacologically treated for depression (JPL) or not (FL and FS). The potential mood mechanism of MIT is based on the fact that music has been shown to have a strong effect on emotions and mood (reviewed in Juslin and Vastfjall, 2008; Koelsch, 2010). Post-stroke depression is associated with greater degree of cognitive impairment and with lower cognitive recovery when controlling for the size of the lesion (Robinson et al., 1986) and music listening leads to better cognitive recovery along with a decrease of depressed and confused mood when compared to stories listening in post-stroke rehabilitation (Särkämö et al., 2008). It was suggested that the power of music on mood could explain a part of the beneficial effects of singing therapies on language recovery. Yet, the effect of music on mood has been shown in rich musical contexts, where subjects listen to, play, or sing real music pieces. In contrast, the musical content of MIT is made of few (usually only two) pitches, its rhythmical structure is poor and there is neither musical syntax nor harmony. A controlled experiment with healthy participants showed that the use of monophonic tones and isochronous beat alone had no significant impact on mood when compared to real musical pieces (Koelsch et al., 2010). Thus, the musical context of MIT might not be sufficient to elicit significant mood changes.

According to the motor-speech hypothesis of MIT’s effect (Zumbansen et al., 2014), the improvements in language production after MIT may be due to the reduction of apraxia of speech, one of the symptoms distinguishing Broca’s aphasia from other aphasic syndromes. It would explain why this specific form of aphasia responds well and consistently to MIT while other forms rarely do (AAN, 1994). In the present study, we did not capture any significant changes in motor-speech agility as measured by one of the sub-tests of the ABA2 (Dabul, 2000). Testing the motor-speech hypothesis of MIT is a challenge due to the lack of quantitative and unanimously accepted assessment tools for apraxia of speech (Ballard et al., 2000). The ABA2 is the best-validated clinical tool currently available. We chose the motor-speech agility subtest because it could be administered to French-speaking participants and we planned to use the norms to decide if changes would be significant. However, ABA2 is a diagnostic tool and it was not validated to detect changes over time in apraxia of speech. Moreover, it is surprising that no significant change was detected on this score when speech accuracy improved on non-trained phrases. For these reasons, we believe that the Diadochokinetic rate subtest of the ABA2 with its current norms is probably not sensitive enough to detect the changes in apraxia of speech with therapy. There is a need to develop sensitive, quantitative assessment methods of apraxia of speech that could be used at the individual level to document intervention-related progress.

Ours is the first study assessing the differential contribution of rhythm and pitch in a version of MIT that preserves all the basic generalization characteristics of the original protocol. As already mentioned, the mood and motor-speech hypotheses had never been assessed. Few speech and language therapies have been tested in such depth with regard to the mechanisms at work in language recovery effects. Given the high inter-individual variability in patients with aphasia, we chose a Latin square cross-over design to be able to compare participants with themselves. Interventions with carry-over effects, as is the case in our study, are theoretically not suited to this design type since periods of wash-out are necessary for the dependent variable to return to baseline before starting the next intervention phase. However, despite the carry-over effects, we were able to capture treatment-related differential improvements. We readily acknowledge that the best experimental design would have been a randomized controlled group study. However, due to the difficulty in recruiting large number of patients with aphasia, especially with the strict selection criteria we applied, it is somewhat unrealistic to investigate the finer aspects of treatment mechanisms in this way.

Finally, the version of MIT that we designed for experimental purposes gave good results at three levels of therapeutic effects with significant improvements on trained, non-trained, and connected speech in the three participants. Combining various verbal materials with a set of repetitive stimuli may constitute an interesting therapeutic mixed principle because it would allow the clinician to evaluate the best language gains achievable by a patient. If only direct effects are obtained in patients with the most severe language impairments, the clinician could focus on real palliative versions of MIT (i.e., that are designed to train a few ready-made useful sentences for the patients’ daily living), and this strategy could be used as a complement to communication-based approaches in speech and language therapy. However, before turning to a fully palliative approach, the mixed principle could allow some patients to show connected speech gains.

## Conflict of Interest Statement

The authors declare that the research was conducted in the absence of any commercial or financial relationships that could be construed as a potential conflict of interest.
